# Pharmacological Inhibition of mTORC1 Prevents Over-Activation of the Primordial Follicle Pool in Response to Elevated PI3K Signaling

**DOI:** 10.1371/journal.pone.0053810

**Published:** 2013-01-11

**Authors:** Deepak Adhikari, Sanjiv Risal, Kui Liu, Yan Shen

**Affiliations:** Department of Chemistry and Molecular Biology, University of Gothenburg, Gothenburg, Sweden; University of Kansas Medical Center, United States of America

## Abstract

The majority of ovarian primordial follicles must be preserved in a quiescent state to allow for the regular production of gametes over the female reproductive lifespan. However, the molecular mechanism that maintains the long quiescence of primordial follicles is poorly understood. Under certain pathological conditions, the entire pool of primordial follicles matures simultaneously leading to an accelerated loss of primordial follicles and to premature ovarian failure (POF). We have previously shown that loss of *Pten* (*phosphatase and tensin homolog deleted on chromosome ten*) in mouse oocytes leads to premature activation of the entire pool of primordial follicles, subsequent follicular depletion in early adulthood, and the onset of POF. Lack of PTEN leads to increased phosphatidylinositol 3-kinase (PI3K)–Akt and mammalian target of rapamycin complex 1 (mTORC1) signaling in the oocytes. To study the functional and pathological roles of elevated mTORC1 signaling in the oocytes, we treated the *Pten*-mutant mice with the specific mTORC1 inhibitor rapamycin. When administered to *Pten*-deficient mice prior to the activation of the primordial follicles, rapamycin effectively prevented global follicular activation and preserved the ovarian reserve. These results provide a rationale for exploring the possible use of rapamycin as a drug for the preservation of the primordial follicle pool, and the possible prevention of POF.

## Introduction

The pool of primordial follicles in the mammalian ovary represents the ovarian reserve, and the female reproductive lifespan depends on the maintenance of the majority of primordial follicles in a quiescent state [1], [2]. A certain number of primordial follicles continuously emerge from this pool and give rise to developing follicles, while the rest of the primordial follicles remain in a dormant but surviving state. It is believed that the persistence of the primordial follicle pool determines the reproductive lifespan in female mammals [2], [3]. Menopause, or ovarian senescence, occurs when the pool of primordial follicles is exhausted [4], [5]. Various pathological conditions can lead to accelerated loss of primordial follicles and the development of premature ovarian failure (POF) [6], [7].

The molecular mechanisms underlying the quiescence and activation of primordial follicles have begun to be elucidated in recent years [2], [3]. From a series of works using genetically modified mouse models, we have shown that phosphatidylinositol 3-kinase (PI3K) signaling in the oocyte controls the survival, loss, and activation of primordial follicles [2], [3]. The functional role of enhanced PI3K signaling in the activation of oocytes was confirmed after it was found that all primordial follicles start to develop simultaneously in mice when *Pten* (*phosphatase and tensin homolog deleted on chromosome ten*), a negative regulator of PI3K, is deleted specifically from oocytes resulting in the global activation of the entire primordial follicle pool [8]. Due to the irreversible nature of primordial follicle activation, the over-activated follicles that are not selected for further development undergo atresia. This eventually leads to the development of POF in the *Pten*-deficient mice [8]. Oocyte-specific deletion of *3′-phosphoinositide-dependent kinase-1* (*Pdk1*), on the other hand, also leads to the premature depletion of the pool of primordial follicles and to development of POF, but in this case the effect is due to a lack of basal level of PI3K activation [9]. This shows that a basal level of PI3K activation in oocytes is essential to maintain the survival of primordial follicles.

It has been shown that PI3K-Akt signaling is constitutively activated in the *Pten*-null oocytes, and this leads to elevated phosphorylation and activation of ribosomal protein S6 (rpS6) [8]. Akt is known to lead to the activation of mammalian target of rapamycin complex 1 (mTORC1) through multiple mechanisms and we have shown *in vitro* that the activation of rpS6 in *Pten*-null oocytes is dependent on mTORC1 signaling [8]. mTORC1 positively regulates cell growth and proliferation by integrating various signals [10], [11]. The heterodimeric complex of tuberous sclerosis complex 1 (TSC1, also known as hamartin) and TSC2 (also known as tuberin) is one of the most important sensors involved in the regulation of mTORC1 activity [10], [11], and mTORC1 is sensitive to the immunosuppressant drug rapamycin [11].

Through a series of genetics experiments, we have shown that increased activation of mTORC1 in mouse oocytes leads to the premature activation of primordial follicles [12]. For example, oocyte-specific deletion of either *Tsc1* [13] or *Tsc2* [14] in mice, which leads to increased levels of active mTORC1, causes the activation of all primordial follicles around the time of puberty and leads to follicular depletion in early adulthood and the associated development of POF. We found that the elevated mTORC1 activity enhanced activation of p70 S6 kinase 1 (S6K1) –rpS6, and that this was responsible for oocyte growth and premature activation of primordial follicles in these mutant mice [12].

Together these observations raise the question of how the PI3K pathway and the mTORC1 pathway cooperate to regulate the activation of primordial follicles in mice. In the current study, we found that treatment of *Pten*-null mice with rapamycin significantly inhibited the global activation of primordial follicles due to the oocyte-specific deletion of *Pten*, which efficiently preserved the primordial follicle pool in the mutant ovaries. Thus, we have shown that PI3K mediates the activation of primordial follicles at least partially through the mTORC1 pathway. More importantly, our results have clinical implications for the prevention of aberrant primordial follicle activation using the existing drug rapamycin, which may be useful for preventing the loss of ovarian follicle reserves after exposure to different ovotoxic agents.

## Results

### Rapamycin Reverses the Over-activation of rpS6 in Oo*Pten* ^−/−^ Oocytes

**Figure 1:**
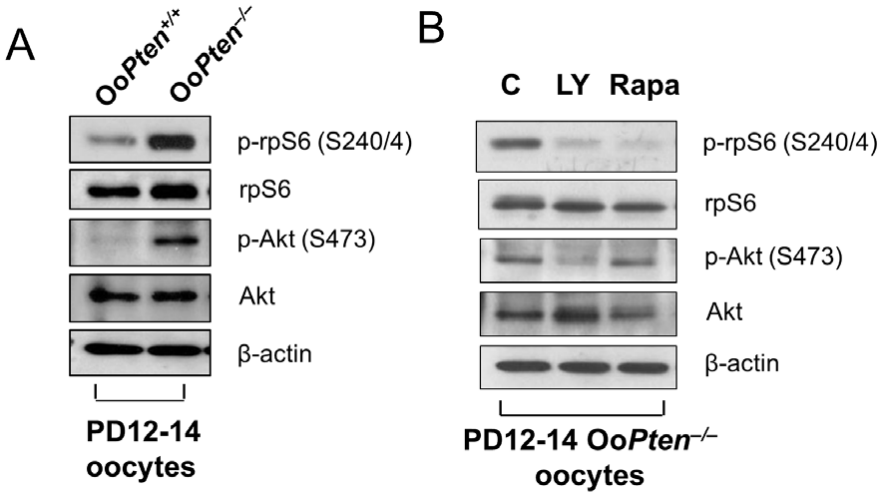
Enhanced Akt-rpS6 activation and *in vitro* inhibition of rpS6 activation in Oo*Pten* ^−/−^ oocytes by rapamycin. (**A**) Comparison of Akt-rpS6 signaling in Oo*Pten* ^−/−^ and Oo*Pten* ^+/+^ oocytes. Oocytes were isolated from ovaries of mice at postnatal day 12–14 and immunoblotting was performed as described in *Materials and Methods*. Loss of PTEN led to enhanced PI3K signaling as indicated by an increase in phosphorylated Akt (p-Akt). The level of phosphorylated rpS6 (p-rpS6) was also increased in Oo*Pten* ^−/−^ oocytes compared with Oo*Pten* ^+/+^ oocytes. Levels of total rpS6, Akt, and β-actin were used as internal controls. (**B**) Activation of rpS6 in Oo*Pten* ^−/−^ oocytes is dependent on mTORC1 signaling. Oocytes were isolated from ovaries of Oo*Pten* ^−/−^ mice at PD 12–14 as described in *Materials and Methods*. Treatment of oocytes with the mTORC1-specific inhibitor rapamycin (Rapa, 50 nM) for 2 h was found to largely suppress levels of phosphorylated rpS6 (p-rpS6), but did not affect the level of phosphorylated Akt (p-Akt). As a control, treatment of Oo*Pten* ^−/−^ oocytes with the PI3K-specific inhibitor LY294002 (LY, 50 µM) for 2 h also largely suppressed levels of phosphorylated rpS6 (p-rpS6), but it also suppressed the level of phosphorylated Akt (p-Akt). This suggests that activation of rpS6 in Oo*Pten* ^−/−^ oocytes is dependent on both PI3K and mTORC1 signaling. Levels of total Akt, rpS6, and β-actin were used as internal controls.

We have previously shown that oocyte-specific deletion of *Pten* (Oo*Pten* ^−/−^) causes global activation of the primordial follicles in the ovaries of the mutant mice [8]. By postnatal day (PD) 23, the entire pool of primordial follicles had been activated in Oo*Pten* ^−/−^ ovaries [8]. PTEN is a major negative regulator of the PI3K/Akt pathway and, therefore, inhibits the phosphorylation and activation of Akt [15]. In this study, we confirmed that the levels of phosphorylation of Akt at residue S473 (p-Akt) and rpS6 at residues S240 and S244 (p-rpS6) were elevated in Oo*Pten* ^−/−^ oocytes (Fig. 1A). It is not known, however, how mTORC1 signaling relates to the PI3K signaling pathway during this process.

mTORC1 promotes cell growth through activation of S6K1 by phosphorylating it on residue T389 [11]. S6K1 is then responsible for the phosphorylation and activation of rpS6 that, together with other ribosomal proteins, leads to enhanced protein translation and ribosome biogenesis [9], [16], [17]. We found that despite the elevated rpS6 phosphorylation (Fig. 1A), phosphorylation of the mTORC1 substrate S6K1 did not increase in Oo*Pten* ^−/−^ oocytes (data not shown).

It is known that mTORC1 is sensitive to rapamycin inhibition [11]. As Akt is known to lead to the activation of mTORC1 through multiple mechanisms, we checked whether or not rpS6 phosphorylation in Oo*Pten* ^−/−^ oocytes was sensitive to rapamycin. We found that although the phosphorylation of S6K1 was not elevated (data not shown), phosphorylation of rpS6 in Oo*Pten* ^−/−^ oocytes was largely inhibited by the mTORC1-inhibitor rapamycin (Fig. 1B). However, rapamycin did not suppress Akt phosphorylation in Oo*Pten^−/−^* oocytes (Fig. 1B). As a control, Oo*Pten* ^−/−^ oocytes were treated with the PI3K-specific inhibitor LY294002 that reduced the phosphorylation of both rpS6 and Akt (Fig. 1B).

These experiments show that mTORC1 is downstream of PI3K-Akt signaling and is responsible for enhancement of protein translation by phosphorylating rpS6 in oocytes.

### Rapamycin Partially Prevents the Premature Activation of Primordial Follicles in Oo*Pten^−/−^* Mice

It has been shown that the phosphorylation and activation of rpS6 in oocytes plays a crucial role in the activation of primordial follicles [9]. We have observed that ablation of *Pten* from oocytes led to hyperphosphorylation of rpS6 (Fig. 1A), which was mediated through the mTORC1 signaling pathway (Fig. 1B). To study the *in vivo* consequences of elevated mTORC1 activity in Oo*Pten^−/−^* mice, we treated these mice with rapamycin from PD 4 to PD 22 (5 mg/kg body weight per day). The mice were sacrificed at PD 23 and the morphology of their ovaries was studied. As controls, Oo*Pten^−/−^* mice were treated from PD 4 to PD 22 with vehicle alone.

**Figure 2:**
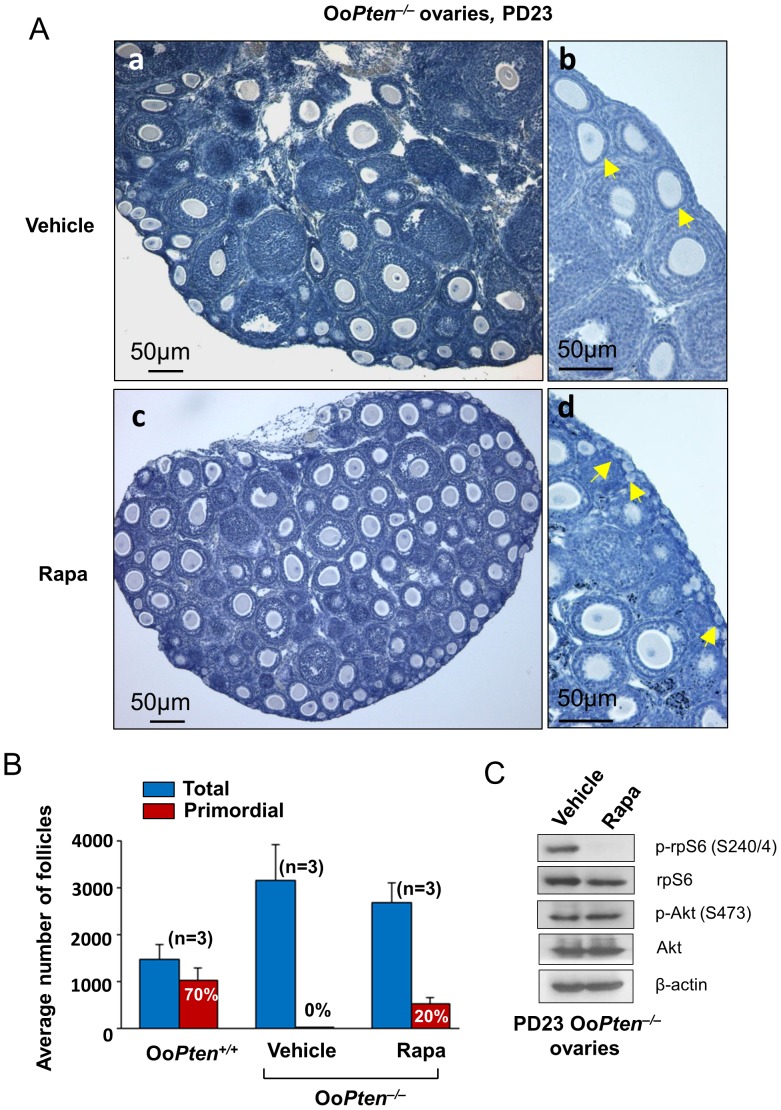
Preservation of the primordial follicle pool in Oo*Pten* ^−/−^ ovaries by rapamycin treatment. (**A**) Prevention of the primordial follicle over-activation in Oo*Pten* ^−/−^ mice by treatment with rapamycin. Rapamycin (5 mg/kg body weight) was injected daily into Oo*Pten* ^−/−^ mice from postnatal day (PD) 4 to PD 22, and the ovaries were collected at PD 23 for morphological analysis. Ovaries from rapamycin-treated Oo*Pten* ^−/−^ mice appeared smaller (c) than the ovaries from vehicle-treated Oo*Pten* ^−/−^ mice (a). Scale bar = 50 µm. Clusters of primordial follicles were seen in rapamycin-treated Oo*Pten* ^−/−^ mice at PD 23 (d, arrows) whereas all primordial follicles were activated in vehicle-treated Oo*Pten* ^−/−^ mice at PD 23 (b, arrows). Scale bar = 50 µm. (**B**) Average numbers of total and primordial follicles in Oo*Pten* ^+/+^, Oo*Pten* ^−/−^ (vehicle-treated), and Oo*Pten* ^−/−^ (rapamycin-treated) ovaries at PD 23. Proportions of primordial follicles ± SEM (relative to the total number of follicles) are also shown. The proportion of primordial follicles in rapamycin-treated Oo*Pten* ^−/−^ ovaries was 20±4.1%, which was smaller than the proportion in the Oo*Pten* ^+/+^ ovaries (70±3.1%). Three mice were used for each experimental group. Rapa, rapamycin. (**C**) Comparison of the rpS6 and Akt phosphorylation levels in the ovaries of vehicle- and rapamycin-treated Oo*Pten* ^−/−^ mice. Rapamycin (5 mg/kg body weight) was injected daily into Oo*Pten* ^−/−^ mice from PD 4 to PD 22, the ovaries were collected at PD 23 and homogenized, and immunoblotting was performed as described in *Materials and Methods*. Rapamycin injection effectively suppressed the level of phosphorylated rpS6 (p-rpS6) without affecting the level of phosphorylated Akt (p-Akt) in the ovaries of Oo*Pten* ^−/−^ mice. Levels of total rpS6, Akt, and β-actin were used as internal controls.

The ovaries in PD 23 Oo*Pten^−/−^* mice were smaller when treated with rapamycin compared to controls receiving vehicle alone (Fig. 2Aa and 2Ac). Notably, the ovaries of the mice that had received rapamycin still contained typical primordial follicles while the ovaries of the mice receiving vehicle alone contained only activated follicles with enlarged oocytes (Fig. 2Ab and 2Ad, arrows).

Follicle counting showed that there was a complete lack of primordial follicles in the control mice (Fig. 2B, Vehicle) while the ovaries of the rapamycin-treated mice still had 20% of their follicles in a primordial state (Fig. 2B, Rapa). Rapamycin treatment did not completely alleviate the premature activation of follicles in the Oo*Pten* ^−/−^ mice because the ovaries of wild type Oo*Pten* ^+/+^ mice had 70% of their follicles in a primordial state (Fig. 2B, Oo*Pten* ^+/+^).

To check rapamycin’s effectiveness in suppressing mTORC1 signaling in Oo*Pten* ^−/−^ ovaries, we measured the level of rpS6 phosphorylation in the ovaries. We found that the phosphorylation of rpS6 in rapamycin-treated Oo*Pten* ^−/−^ ovaries was suppressed (Fig. 2C), but this was not mediated through the suppression of PI3K signaling because rapamycin did not suppress the phosphorylation of Akt (Fig. 2C).

These results clearly show that the hyperphosphorylation of rpS6 and the over-activation of the primordial follicle pool in Oo*Pten* ^−/−^ mice were mediated in large part through the activation of the mTORC1 pathway. At the same time, these results also imply that suppression of mTORC1 activity in oocytes is necessary for the preservation of primordial follicles in a quiescent state.

## Discussion

In the current study, we treated Oo*Pten^−/−^* mice with the mTORC1 inhibitor rapamycin and showed that a significant amount of the ovarian reserve in Oo*Pten^−/−^* mice is retained, which rescued the female mice from POF that is caused by excessive activation of the primordial follicle pool. These findings are in agreement with our previous report that rapamycin treatment prevented the over-activation of primordial follicles in mice lacking the *Tsc1* gene in their oocytes (Oo*Tsc1* ^−/−^ mice) [13]. Thus, our results show that the suppression of mTORC1 signaling plays an important physiological role in the maintenance of the dormant pool of primordial follicles, which is essential for preserving the length of female reproductive life.

In our earlier study, we showed that although all primordial follicles in Oo*Tsc1* ^−/−^ mice were activated, rapamycin could prevent such over-activation of primordial follicles in Oo*Tsc1* ^−/−^ mice [13]. In the current study, 20% of the primordial follicles in Oo*Pten* ^−/−^ mice (Fig. 2B, Rapa) were maintained in a quiescent state after rapamycin treatment. This is because enhanced mTORC1 signaling was the only factor behind the global activation of primordial follicles in the Oo*Tsc1* ^−/−^ mice [13] whereas both PI3K signaling and mTORC1 signaling were upregulated in the oocytes of the Oo*Pten* ^−/−^ mice and jointly enhanced follicular activation (Fig. 1B). Because rapamycin is an mTORC1-specific inhibitor, it only suppressed mTORC1 signaling and not PI3K-Akt signaling (Fig. 1B and 2C).

Chemotherapeutic treatments often induce POF due to the loss of the primordial follicle pool, but whether or not these treatments directly eliminate primordial follicles has not been clearly shown [18], [19]. It has, however, been proposed that the reduction in the primordial follicle pool can arise indirectly as a consequence of the destruction of growing follicles by the chemotherapeutic agents [20]. The granulosa cells of the growing follicles produce anti-Müllerian hormone (AMH) that inhibits further primordial follicle activation [21]. Thus, a sudden loss of the already growing follicles by chemotherapy might result in increased activation of primordial follicles and their recruitment into the pool of growing follicles [18].

Several environmental chemicals that cause POF in rodent models have been shown to enhance PI3K and mTORC1 signaling pathways and lead to over-activation of primordial follicles [22], [23]. The polycyclic aromatic hydrocarbon 7,12-dimethylbenz-[a]anthracene (DMBA) is known to cause atresia in developing follicles and over-activate primordial follicles through enhanced activation of PI3K and mTORC1 signaling in rodent models of cancer [22]. Another ovotoxic chemical, 3-Methylcholanthrene (3MC), specifically targets developing follicles for destruction and induces premature activation of primordial follicles through the PI3K and mTORC1 signaling pathways [23]. These observations suggest that manipulation of the signaling pathways involved in follicular development can play a significant role in female reproductive health. Thus a better understanding of the molecular mechanism of primordial follicle activation may have broad physiological and clinical implications.

In summary, in this study we have shown that rapamycin treatment can prevent the complete loss of primordial follicles that would otherwise occur in mice with an oocyte-specific deletion of *Pten.* The implication of these results is that rapamycin may be clinically useful in maintaining at least a portion of the primordial follicle pool in the ovaries of women who are otherwise at risk for POF. This is especially relevant for women who are being treated with ovotoxic chemotherapeutic agents and current work in our lab seeks to address the ability of rapamycin to function in such cases. Recently, chronic treatment with mTOR inhibitors has been reported to increase the risk of menstrual-cycle disturbances and ovarian cysts in women [24] and to lead to reduced ovulation in mice [25]. Thus, further studies are required to evaluate such possible adverse effects of rapamycin on reproductive abnormalities in humans.

## Materials and Methods

### Mice

*Pten^loxP/loxP^* mice [8] with a C57BL/6J genomic background were crossed with transgenic C57BL/6J mice that carried a Cre recombinase under the control of a growth differentiation factor 9 (*Gdf-9*) promoter [26]. After multiple rounds of crossing, we obtained homozygous mutant female mice lacking *Pten* in their oocytes (Oo*Pten* ^−/−^ mice). Control mice that do not carry the Cre transgene are referred to as Oo*Pten* ^+/+^ mice. The mice were housed under controlled environmental conditions with free access to water and food. Illumination was on between 0600 and 1800 h.

Ethics Statement: Experimental protocols were approved by the regional ethical committee of the University of Gothenburg, Sweden.

### Reagents, Antibodies, and Immunological Detection Methods

The rabbit polyclonal antibodies to Akt, phospho-Akt (S473) and phospho-rpS6 (S240/4) were obtained from Cell Signaling Technologies (Beverly, MA). Mouse monoclonal antibody to rpS6 was purchased from Santa Cruz Biotechnology Inc. (Santa Cruz, CA). Western blots were carried out according to the instructions of the suppliers of the different antibodies and visualized using the ECL Plus Western Blotting Detection System (Amersham Biosciences, Uppsala, Sweden).

### Quantification of Ovarian Follicles and Histological Analysis

Quantification of ovarian follicles was performed as previously described [8]. Briefly, ovaries were fixed in 4% paraformaldehyde, dehydrated, and embedded in paraffin. To count the numbers of follicles, the paraffin-embedded ovaries were cut into 8-µm sections and stained with hematoxylin for morphological observation. Ovarian follicles at different stages of development, including primordial follicles, transient follicles containing enlarged oocytes surrounded by flattened pregranulosa cells, type 3b, type 4, type 5, and type 6 follicles were counted in all sections of an ovary based on the standards established by Pedersen and Peters [27]. Follicles that contained oocytes with clearly visible nuclei were scored in each section as previously reported [28]. Careful morphological analysis reduced the incidence of counting the same follicle twice or of missing a follicle.

### Isolation of Oocytes from Postnatal Mouse Ovaries and Inhibitor Treatment

Isolation and lysis of oocytes were performed as previously described [8]. Oocytes were isolated from ovaries of 12- to 14-day-old mice. For each experiment, material from 3–5 mice was used per lane and approximately 20 µg of protein was loaded for immunoblotting. Oocytes were incubated with 50 µM of the PI3K-specific inhibitor LY294002 (EMD Biosciences, San Diego, CA) or 50 nM of the mTORC1-specific inhibitor rapamycin (EMD Biosciences, San Diego, CA) for 2 h before being lysed in the lysis buffer.

### *In Vivo* Treatment of Mice with Rapamycin

Rapamycin was dissolved in a vehicle containing 5.2% Tween 80 (Sigma-Aldrich Sweden AB, Stockholm, Sweden) and 5.2% polyethylene glycol 400 (Sigma-Aldrich Sweden AB, Stockholm, Sweden). For daily *intraperitoneal* injection of the mice, a dose of 5 mg/kg body weight was used. The mice were injected daily from PD 4 to PD 22 and were killed at PD 23. The ovaries were then fixed in 4% paraformaldehyde, dehydrated, and embedded in paraffin. For morphological observation and follicle counting, paraffin-embedded ovaries were cut into 8-µm sections and stained with hematoxylin. Mice injected with vehicle alone were used as controls. All experiments were repeated at least three times.
